# Design Challenges for HIV-1 Vaccines Based on Humoral Immunity

**DOI:** 10.3389/fimmu.2014.00335

**Published:** 2014-07-16

**Authors:** Neil S. Greenspan

**Affiliations:** ^1^Department of Pathology, Case Western Reserve University, Cleveland, OH, USA

**Keywords:** genomic diversity, immune escape, broadly neutralizing antibody, non-neutralizing antibody, vaccine-guided B cell evolution, immunogen design, regulatory CD8^+^ T cells, vectored immunoprophylaxis

## Introduction

Human Immunodeficiency Virus-1 (HIV-1) presents profound challenges to vaccine developers. Potential hurdles of relevance to the design of an effective HIV-1 vaccine based on humoral immunity include: (1) the exceptional rate of mutation of the viral genome due to an error-prone polymerase (i.e., reverse transcriptase) (1), (2) a relatively high rate of genomic recombination (2, 3), (3) both within-host and between-host evolution (4), (4) extensive glycosylation of the chief antigenic target (gp120) recognized by potentially protective antibodies (5, 6), (5) immunodominance of regions of the envelope glycoprotein that display a high degree of primary structure diversity (7) favoring the generation of neutralizing antibodies of narrow breadth, (6) a relatively low density of antigen spikes on the virion surface (8, 9) thereby minimizing multivalent antibody binding and perhaps raising the threshold affinity required for potent neutralization by at least some antibodies, (7) the ability of HIV to infect CD4^+^ T cells and other cells critical to functioning of the immune system, and deplete the numbers of these cells, and (8) perhaps most insidious, activation of CD4^+^ T cells, which is necessary for the generation of potent broadly neutralizing antibodies in response to immunization may simultaneously increase the number of cells susceptible to infection by HIV (10, 11).

The scale of the antigenic diversity characterizing the HIV-1 envelope molecules, which are the primary targets of antibody-mediated immunity, is truly daunting. According to Korber et al. (1), the HIV-1 viral genomes in one infected individual encompass the same approximate extent of nucleotide sequence diversity that is exhibited by the worldwide population of influenza A viral genomes over the course of a year.

While most HIV transmission events appear to trace back to a single virus, up to a quarter of infections may involve infection by two to five viruses (12). So, even if vaccine immunization elicits a robust antibody response, the probability that the antibodies circulating in the blood of a vaccinated individual will effectively neutralize or otherwise mediate immunity against all of the viruses mediating infection will be greatest if those antibodies are broadly neutralizing. In addition, due to rapid within-host evolution, substantially different viruses may be infecting different individuals in a large population. Therefore, antibodies generated by vaccination will need to be broadly neutralizing to achieve high levels of vaccine efficacy.

## Unique Features of Broadly Neutralizing Antibodies for HIV-1

Investigators interested in HIV-1 vaccines based on humoral immunity have established that most neutralizing antibodies in most patients neutralize a limited range of strains, primarily those to which the individual patient was exposed or closely related strains. In contrast, only about 25–30% of patients synthesize potent and broadly neutralizing antibodies (pbnAb) and typically only after 2–4 years following the initiation of infection. This time interval for the development of protective antibodies is exceptionally long.

The extent of somatic hypermutation focused on the rearranged immunoglobulin (Ig) genes encoding the heavy and light chain variable domains and that has been associated with broad neutralizing activity against HIV-1 is also extraordinary. Ig genes encoding typical anti-pathogen antibodies from a mature immune response may exhibit on the order of 15–20 mutations in the gene segments encoding the heavy chain variable domain. In contrast, many pbnAb for HIV-1 have 40–100 somatic mutations in the genes encoding the heavy chain variable domain. It seems plausible to infer that a truly exceptional extent of Ig gene evolution is contributory to and possibly essential for the generation of at least some pbnAb. This inference is supported by the recent findings that many pbnAb contain many heavy chain V domain-encoding mutations in framework regions and these mutations, at both contact and non-contact positions, are necessary for both high potency and great breadth of neutralization activity (13). Although, the high number of variable domain mutations in pbnAb may also reflect in part the rapid pace of within-host HIV evolution, this possibility does not obviously account for the typical and rather extended time frame observed for the development of pbnAb.

In either case, it will also be important to study the critical role of follicular helper CD4^+^ cells (14) in driving proliferation as well as somatic hypermutation and affinity maturation in germinal center B cells. Another useful focus for future investigation is based on the recent insights regarding the neutralizing activities of glycan-specific Ab (15).

Thus, it is fair to question whether the standard approach to clinical vaccination of administering the same immunogen several times is likely to be effective in the case of HIV-1. A number of major laboratories investigating this problem have already begun fashioning novel approaches in which the ultimate overall goal is likely to be immunization with a series of related but distinguishable HIV-1-derived envelope molecules that guide the evolutionary trajectory for the genes encoding the variable (V) domains of the corresponding neutralizing antibodies (16). Such a scheme would appear to recapitulate to some extent the *in vivo* process that occurs for B cells in infected patients. The logistics of vaccine delivery entailed by this sort of immunization scheme may be more challenging than for any previously successful clinical vaccine.

A crucial feature of this approach of guiding B cell evolution to the production of pbnAb reflects the troubling fact that the epitope recognized by a protective Ab at the end of the process may not be bound by the B cell receptors (BCR) of the germline B cells from which such antibodies will ultimately be derived by clonal evolution (16). Therefore, vaccine developers have begun working to identify antigens suitable for the activation of the B cells expressing germline BCRs that can serve as the ancestral sequences for pbnAb.

This sort of approach is undoubtedly made more plausible by recent and impressive advances in structure-guided methods for designing vaccine immunogens to contain specific epitopes (16, 17). The results of Dey et al. and Jardine et al. suggest the potential of these new techniques for eliciting antibody responses of desired specificity for the HIV-1 envelope protein.

## Remaining Questions Regarding Immunity Elicited by Vaccine-Guided B Cell Evolution

Nevertheless, it remains to be determined: (1) if identification of monoclonal pbnAb can lead to designed vaccine immunogens that reliably elicit protective polyclonal serum or mucosal antibodies, (2) how many sequential immunogens will be required to reliably guide the evolutionary trajectory to pbnAb in the great majority of vaccine recipients, (3) how many total immunizations will be needed, (4) what intervals between immunizations would achieve the optimal balance between immunogenicity and a timeframe for elicitation of protective responses compatible with public health goals, and (5) how much expense such a scheme, implemented on a mass scale, will entail.

The preceding strategy is based on the assumption that the most viable approach to generating effective immunity to HIV-1 is to elicit pbnAb by an active humoral immune response. There is evidence that non-neutralizing antibodies may be able to contribute to immunity to the virus (18–20), but it appears that only a minority of investigators focusing on HIV vaccines based on humoral immunity are persuaded that such immunity can be of sufficient potency to protect vaccine recipients in the absence of pbnAb. It is of importance of determine if elicitation of non-neutralizing antibodies to HIV-1 antigens is a feasible path to successful protection in a high percentage of vaccine recipients.

## Regulating CD4^+^ T Cells as a Path to Immunity to HIV-1

A counter-intuitive approach to vaccine development, although not based in elicitation of humoral immunity, merits brief discussion. Since HIV-1 infects CD4^+^ T cells (which are crucial for the sorts of humoral immune responses we address above), and susceptibility to HIV-1 is increased after activation through the T cell receptor, Lu et al. (10) reasoned that an immunogen able to diminish responses of CD4^+^ T cells might actually reduce susceptibility of a recipient of that immunogen to HIV infection. These authors demonstrated that administration of an oral vaccine consisting of an inactivated simian immunodeficiency virus (SIVmac239) plus the tolerance-inducing commensal bacterium *Lactobacillus plantarum* to macaques protected them from subsequent intrarectal challenge. CD8^+^ T cell-depleting antibodies confirmed the necessary role of CD8^+^ regulatory T cells to this unusual form of vaccine-mediated immunity to infection.

In the macaque model of SIV infection described by Lu et al., the beneficial effect of reduced responsiveness by CD4^+^ T cells was mediated by CD8^+^ T cells recognizing antigens presented by non-classical MHC class I molecules. It will obviously be of interest to determine if the same cell type could operate similarly in humans, assuming the phenomenon is reproducible across species. Another important question that merits investigation is whether this approach to vaccination is effective in eliciting protection against infection for virus entry by other routes. Even if in humans, a vaccine targeting CD8^+^ T cells recognizing antigens presented by non-classical MHC class I molecules were able to provide protection from HIV infection, it would not necessarily rule out the possibility of manipulating standard CD4^+^ regulatory T cells to add to any benefit associated with CD8^+^ regulatory T cells.

## Vectored Immunoprophylaxis as an Approach to Humoral Immunity

Another approach to generating humoral immunity to HIV-1 is based not on induction of active B cell immunity but on vectored immunoprophylaxis (21, 22), In this strategy, a viral vector (e.g., adeno-associated virus, AAV) is used to infect or otherwise insert genes encoding intact potent broadly neutralizing antibodies into host cells *ex vivo* with subsequent implantation or *in vivo*. This scheme provides arguably passive immunity in that there is no administration of an immunogen related to HIV and no elicitation of an immune response involving host B lymphocytes, as usually defined. However, the production of antibodies is active in the host and no already synthesized antibodies are directly infused. Early studies in animal models have demonstrated proof of principle for using vectored immunoprophylaxis to confer robust protection to recipient animals challenged with significant doses of virulent HIV by clinically relevant routes (21, 22).

Of course, applying vectored immunoprophylaxis on a mass scale as an alternative to standard vaccination also requires addressing so far unanswered questions. Who should receive the treatment and at what age? Is a single administration of the vector sufficient for long-lasting antibody production and protection? Are periodic administrations of the vector needed to maintain persistent protective immunity? Could HIV evolve so as to escape one or even multiple pbnAb generated via vectored immunoprophylaxis? If antibody produced by the vector produced undesirable side-effects, how could synthesis be abrogated in a timely manner? Will the genetic vectors persist in treated patients for periods and in ways that cause unwelcome side-effects? Can proponents of vectored immunoprophylaxis produce data that will assure the FDA that these safety concerns have been adequately addressed?

## Conclusion

Due to a constellation of attributes including enormous genomic and antigenic sequence diversity, rapid evolution, and immunity-subverting structural features of key antigens associated with HIV-1, it is one of the most challenging pathogens vaccine developers have confronted to date. Technological advances in cloning Ig genes from individual human B lymphocytes, generating human monoclonal antibodies, and designing immunogens to express one or a limited number of epitopes have been extraordinary and rapid. These advances make plausible a vaccination scheme centered on the notion of using a series of related but non-identical immunogens to guide the process of B cell evolution through repeated rounds of somatic hypermutation leading to affinity maturation and acquisition of broadly neutralizing activity. However, numerous scientific and logistical challenges remain to be addressed before such a scheme could be implemented on a public health scale. Therefore, alternative strategies to generating protective immunity to HIV-1, such as vectored immunoprophylaxis or induction of regulatory responses intended to reduce activation of CD4^+^ T cells, remain worthy of thorough exploration.

## Conflict of Interest Statement

The author declares that the research was conducted in the absence of any commercial or financial relationships that could be construed as a potential conflict of interest.
